# Non-invasive assessment of hepatic involvement in common variable immunodeficiency and agammaglobulinemia using enhanced liver fibrosis score and shearwave elastography

**DOI:** 10.3389/fimmu.2026.1857089

**Published:** 2026-06-24

**Authors:** Maja Stojanovic, Milica Stojkovic-Lalosevic, Sanja Stankovic, Rada Miskovic, Jelena Ljubicic, Ivan Rankovic, Andrej Pesic, Sanja Dragasevic Vucicevic, Gordana Petrovic, Branka Bonaci-Nikolic

**Affiliations:** 1 Clinic of Allergy and Immunology, University Clinical Center of Serbia, Belgrade, Serbia; 2 Faculty of Medicine, University of Belgrade, Belgrade, Serbia; 3 Clinic of Gastroenterology and Hepatology, University Clinical Center of Serbia, Belgrade, Serbia; 4 Center for Medical Biochemistry, University Clinical Center of Serbia, Belgrade, Serbia; 5 Faculty of Medical Sciences, University of Kragujevac, Kragujevac, Serbia; 6 Clinic of Hematology, University Clinical Center of Serbia, Belgrade, Serbia; 7 Institute for Health Care of Mother and Child of Serbia Dr. Vukan Cupic, Belgrade, Serbia

**Keywords:** agammaglobulinemia, common variable immunodeficiency, elastography, ELF score, liver fibrosis, enteropathy

## Abstract

**Background:**

Early detection of hepatic involvement in common variable immunodeficiency (CVID) and agammaglobulinemia (AGA) remains hampered by the absence of validated non-invasive biomarkers. Although the Enhanced Liver Fibrosis (ELF) score is well established across different liver diseases, its role in CVID/AGA remains undefined. We aimed to assess hepatic involvement using ELF and shearwave elastography (SWE), and to evaluate their correlation and agreement.

**Methods:**

This cross-sectional study included 22 patients with CVID and 9 with AGA without previously known/documented liver fibrosis. The ELF score was calculated from serum hyaluronic acid (HA), tissue inhibitor of metalloproteinases-1, and N-terminal propeptide of type III collagen. Liver stiffness (LS) was measured using SWE. False discovery rate was controlled using the Benjamini–Hochberg method.

**Results:**

According to SWE, 64.5% of patients had elevated LS, whereas increased ELF values corresponding to intermediate and severe/advanced fibrosis were present in 58% and 35.5%, respectively. There was a statistically significant agreement (weighted Cohen’s κ=0.324; p=0.002) along with a significant correlation between ELF and LS (p=0.01/pbh=0.05). In addition, ELF values were also positively associated with AST (p<0.001/pbh<0.001) and ALP (p=0.003/pbh=0.03). Among individual ELF components, HA showed consistent associations with LS and liver enzymes, including ALT, AST, and γGT (p=0.01/pbh=0.05; p<0.001/pbh<0.001; p=0.004/pbh=0.04, respectively).

**Conclusion:**

Our exploratory findings suggest that combining ELF and SWE may provide a promising non-invasive approach for liver assessment, potentially reflecting different but complementary aspects of hepatic involvement in CVID and AGA; however, their clinical utility requires validation in larger, prospective, multicenter studies with histopathological correlation to define disease-specific thresholds.

## 1 Introduction

Common variable immunodeficiency (CVID) is the most common symptomatic primary antibody deficiency, characterized by marked clinical heterogeneity, encompassing both infectious susceptibility and a wide range of non-infectious complications (1, 2). Among these, liver disease is increasingly recognized as a significant contributor to morbidity and disease-related mortality (3–6). Approximately 50% of patients with CVID exhibit some form of hepatic involvement during the disease course, with reported prevalence rates varying depending on the diagnostic procedures applied (7, 8). The clinical spectrum of CVID-associated liver disease (CVID-L) is broad, ranging from mildly elevated liver enzymes to portal hypertension, which may progress to hepatic decompensation and liver failure (3, 4, 8, 9). Nodular regenerative hyperplasia (NRH), a subgroup of porto-sinusoidal vascular disorder (PSVD), represents the most frequently observed histopathological hepatic pattern of CVID-L and is widely regarded as a non-cirrhotic cause of portal hypertension (4, 10, 11). Despite its clinical relevance, the pathogenesis of NRH in CVID remains poorly understood, and disease-specific diagnostic guidelines and therapeutic strategies are currently lacking (12). Beyond mild cholestatic enzyme elevations, increased liver stiffness (LS) and splenomegaly, particularly spleen size exceeding 16 cm in diameter, have been proposed as surrogate markers of evolving portal hypertension (9, 13). CVID-enteropathy (CVID-E) may contribute to hepatic involvement through shared immune dysregulation, chronic inflammation, and increased intestinal permeability with bacterial translocation mediated liver inflammation (7, 14).

Available evidence on the role of imaging in CVID-related portal hypertension remains conflicting. Whereas some reports demonstrate elevated transient elastography (TE) values in the absence of ultrasound-detectable portal hypertension (4), other studies question the ability of TE to reliably identify early or pre-fibrotic disease, highlighting substantial variability across published data (15–17). Owing to the heterogeneity of CVID-L, early detection of hepatic involvement remains challenging despite the use of a multimodal diagnostic approach. In addition, reliable and easily applicable serum biomarkers for early disease detection are still lacking.

The Enhanced Liver Fibrosis (ELF) score incorporates serum levels of hyaluronic acid (HA), the N-terminal propeptide of type III collagen (PIIINP), and tissue inhibitor of metalloproteinases-1 (TIMP-1). It provides a non-invasive assessment of extracellular matrix (ECM) turnover by capturing the dynamic interplay between fibrogenic and fibrolytic activities and has been validated across a range of chronic liver diseases (18, 19). To date, only TIMP-1 has been investigated in CVID, predominantly in the context of immune dysregulation and granulomatous-lymphocytic interstitial lung disease (GLILD) (20). However, the potential role of other ECM biomarkers, including HA and PIIINP, and the ELF score, as markers of hepatic involvement in CVID has not been systematically explored.

Given the central role of immune dysregulation, endothelial injury, and consequent aberrant tissue remodeling in CVID-associated complications (21), biomarkers reflecting ECM turnover may provide additional insight into early hepatic involvement. The aim of this study was to assess the potential of HA, PIIINP, and TIMP-1, both individually and within the ELF score, in the evaluation of hepatic involvement in CVID and AGA, and to evaluate their correlation and agreement with LS obtained by shearwave elastography (SWE).

## 2 Materials and methods

### 2.1 Study design and participants

This cross-sectional exploratory study included 31 adult participants, comprising 22 patients with a confirmed diagnosis of CVID and 9 patients with AGA. Diagnosis of CVID was made according to the European Society for Immunodeficiencies (ESID) criteria (22). Patients with AGA were defined by profoundly reduced or absent circulating B cells and markedly decreased serum immunoglobulin (Ig) levels, with genetic confirmation performed when available (1). None of the patients had previously known/documented fibrosis, and liver function tests were performed concurrently with LS measurement and ELF assessment. All patients received regular immunoglobulin replacement therapy (IRT). Within the CVID cohort, particular emphasis was placed on the enteropathy due to its reported association with hepatic manifestations. The CVID-E group was characterized by the presence of diverse clinical manifestations, including celiac disease-like or inflammatory bowel disease-like pathology, as determined by radiographic, endoscopic, and histological findings (23, 24). For comparative purposes, an age- and sex-comparable cohort of patients (n=42) with metabolic dysfunction-associated steatotic liver disease (MASLD), defined according to international consensus criteria (25), was additionally included in the study. MASLD was selected as one of the most extensively studied chronic liver diseases with well-characterized SWE parameters, including LS, tissue attenuation imaging (TAI), and tissue scatter distribution imaging (TSI) (26).

### 2.2 Standard clinical and laboratory assessment

All participants underwent comprehensive clinical evaluation and venous blood sampling for assessment of biochemical parameters, platelet count, and serum fibrosis markers. Serum immunoglobulin G (IgG) was measured on the day of the scheduled IRT, corresponding to trough concentrations and in accordance with routine clinical practice for monitoring treatment adequacy**;** Biochemical analyses included measurements of liver enzymes (alanine aminotransferase (ALT), aspartate aminotransferase (AST), alkaline phosphatase (ALP), and gamma-glutamyltransferase (γGT); coagulation parameters (international normalized ratio (INR), albumin and fibrinogen; and lipid profile components (high-density lipoprotein cholesterol (HDL), low-density lipoprotein cholesterol (LDL), and triglycerides). Abdominal ultrasound examination and SWE were performed within the same session on the day of the scheduled IRT, using the same ultrasound system.

### 2.3 Evaluation of liver fibrosis-related biomarkers

Serum concentrations of liver fibrosis-related biomarkers, including HA, PIIINP, and TIMP-1 were determined using chemiluminescent immunoassays on the ADVIA Centaur XP analyzer (Siemens Healthineers, Erlangen, Germany) with manufacturer-supplied reagents and calibrators. All marker concentrations were expressed in ng/mL. The ELF score was computed automatically by the system using the validated proprietary formula: ELF=2.278+0.851∗ln(HA)+0.751∗ln(PIIINP−1)+0,394∗ln(TIMP−1), and expressed as a unitless numerical value. Cut-off values for the ELF score were applied according to the manufacturer’s recommended thresholds, which were previously validated in a study including 400 healthy controls and patients across a range of chronic liver diseases (18, 27). ELF reference values in healthy individuals ranged from 6.7 to 9.8. Predefined cut-off values were established at 7.7 for high-sensitivity exclusion of fibrosis, 9.8 for high-specificity identification of significant fibrosis, and 11.3 for cirrhosis (27). Accordingly, values<7.7 were considered indicative of no or minimal fibrosis (F0), 7.7–9.8 of intermediate (F1), 9.9–11.3 of severe fibrosis (F2), and >11.3 of advanced fibrosis/cirrhosis (F3).

### 2.4 Liver stiffness measurement

Liver stiffness was assessed using SWE (Samsung RS80A) by a trained hepatologist. Measurements were conducted under fasting conditions, with patients in supine position and the right arm maximally abducted. For each participant, at least 10 valid measurements were obtained, and the median LS value (expressed in kilopascals, kPa) was used for analysis. Only measurements with an interquartile range (IQR) of <30% were considered valid, in accordance with established guidelines. Liver fibrosis stages were defined according to proposed cut-off values for LS measured by two-dimensional SWE as follows: <6.5 kPa for F0 (absent or mild fibrosis), 6.5–9.7 kPa for F1 (significant fibrosis), 9.8–13.1 kPa for F2 (advanced fibrosis), and **>**13.1 kPa for F3 (cirrhosis) (28). Additionally, Tissue Attenuation Index (TAI), reflecting hepatic ultrasound signal attenuation, and Tissue Scatter Imaging (TSI), measuring parenchymal microstructural heterogeneity, were also provided.

### 2.5 Statistical analysis

Clinical, laboratory, and SWE parameters, including LS measurements, were compared between CVID and AGA groups. Correlations between serum fibrosis markers and LS, IgG levels, ALT, AST, ALP, γGT, platelet count, and fibrinogen levels were assessed. Finally, considering the increased risk of hepatic involvement, patients with CVID-E were compared with patients with CVID without enteropathy. Given the limited sample size and the rarity of CVID-E, this subgroup analyses were considered exploratory and hypothesis-generating. Statistical analyses were performed using EZR (Easy R, version 1.68). Categorical variables are presented as counts and percentages. Continuous variables are reported as mean ± standard deviation (SD) for normally distributed data, or as median with interquartile range (IQR) for non-normally distributed data. Group comparisons were conducted using Fisher’s exact test or the chi-square test for categorical variables, and two-sample t-test or Mann–Whitney U test for continuous variables, as appropriate. Pearson’s correlation coefficient was used for normally distributed continuous variables, and Spearman’s rank correlation coefficient for non-normally distributed variables. P values were adjusted for multiple comparisons using the Benjamini–Hochberg procedure to control the false discovery rate. Weighted Cohen’s kappa with linear weighting was calculated to assess agreement between ordinal ELF and SWE categories. All tests were two-tailed, and a p-value<no><</no> 0.05 was considered statistically significant. Effect sizes were calculated for all comparisons to provide an estimate of the magnitude of observed differences. Rank-biserial correlation (r) was used for Mann–Whitney U tests, Cohen’s d for independent-samples t-tests, and Cramér’s V for categorical variables. Approximate 95% confidence intervals were determined for all effect size estimates.

## 3 Results

### 3.1 Demographic and clinical characteristics of the study population

Thirty-one patients without previously known/documented liver fibrosis were enrolled in this study, with the majority diagnosed with CVID (n=22; 71%). The median age was 39 years. Completely normal liver tests were obtained in 87% patients. Median liver enzyme levels (AST, ALT, ALP, γGT), IgG concentrations, and platelet counts were all within the reference range (**Table 1**).

**Table 1:** Demographic and clinical patients’ characteristics. .

	CVID	AGA	*P-value*	Effect-size (95% CI)
Diagnosis (N, %)	22 (71%)	9 (29%)		
Age (median, IQR)	56 (44.25-58)	37.5 (30.25-60.25)	0.25	0.55^r^ (0.13-0.85)
Male gender (%)	13 (59%)	8 (88.9%)	0.20	-0.28^v^
Biochemical parameters				
ALT U/L (median, IQR)	24 (15.5-28.5)	32 (15.5-45)	0.37	-0.06 (-0.42-0.57)^r^
AST U/L (median, IQR)	26 (18.5-30)	24 (15-29.75)	0.77	0.29 (-0.73-0.29)^r^
γGT U/L (median, IQR)	22 (16-28)	34 (19.25-63.75)	0.38	-0.35 (-0.88-0.41)^r^
ALP U/L (median, IQR)	93 (75-111)	93 (74-121)	1.00	0.01 (-0.59-0.59)^r^
Fibrinogen g/L (mean, SD)	3.93 (0.87)	3.94 (0.93)	0.97	0.01 (-1.03-1.00)^d^
IgG g/L (median, IQR)	7.71 (3.7-9.49)	6.41 (5.9-8.85)	0.72	0.26 (-0.48-0.46) ^r^
Platelets x 10^9^ (median, IQR)	209 (147-300)	238 (191.5-286.5)	0.49	-0.17 (-0.61-0.32)^r^
Liver stiffness parameters				
LS (median, IQR)	6.7 (5.8-8.45)	7.1 (6.5-7.2)	0.97	-0.02 (-0.51-0.49)^r^
TAI (mean, SD)	0.61 (0.08)	0.59 (0.05)	0.59	0.29 (-0.88-1.57)^d^
TSI (mean, SD)	91.69 (8.02)	84.29 (2.22)	0.14	1.08 (-0.29-2.23)^d^
Fibrosis biomarkers				
HA (ng/mL) median (IQR)	21.07 (11.7-91.6)	62.9 (33.3-98.5)	0.24	-0.28 (-0.75-0.23)^r^
TIMP-1 (ng/mL) median (IQR)	19.45 (10.8-159.6)	119.6 (13-222.5)	0.27	-0.26 (-0.65-0.19)^r^
PIIINP (ng/mL) median (IQR)	196.8 (18.8-239.6)	22.9 (12-159.7)	0.16	0.33 (-0.79-0.1)^r^
ELF score (mean, SD)	9.23 (1.73)	9.76 (1.36)	0.37	-0.34 (-0.42-1.14)^d^

Effect sizes are presented as rank-biserial correlation (r) for Mann–Whitney U tests, Cohen’s d (d) for t-tests, and Cramér’s V (V) for categorical variables.

CVID-common variable immunodeficiency, AGA-agammaglobulinemia, 95% CI-95% confidence interval, ALT-alanine aminotransferase, IQR-interquartile range, AST-aspartate aminotransferase, γGT-gamma-glutamyl transferase, ALP-alkaline phosphatase, SD-standard deviation, IgG-immunoglobulin G, LS-liver stiffness measured by shear wave elastography, TAI-tissue attenuation imaging, TSI-tissue scatter imaging, HA-hyaluronic acid, TIMP-1-tissue inhibitor of matrix metalloproteinase, PIIINP-N-terminal pro-peptide of type III collagen, ELF-enhanced liver fibrosis score.

Normal values: AST 0–37 U/L, ALT 0–41 U/L, ALP 40–120 U/L, gGT 0–49 U/L, fibrinogen 2.2-5.5 g/L, IgG 5.5-16.3 g/L, platelets 150-450x10^9^/L.

### 3.2 SWE-derived liver stiffness and tissue characteristics

Liver stiffness values ranged from 5.40 to 20.00 kPa (median 7.1; IQR 5.8–8.4). Based on SWE-derived LS, 64.5% of patients with CVID and AGA exhibited elevated values corresponding to fibrosis stages F1–F3. When analyzed according to diagnosis, no statistically significant difference was observed between CVID and AGA groups [median 6.7 (QR 5.8–8.45) vs. 7.1 (IQR 6.5–7.2), respectively; p=0.97]. Median and mean values of LS measurements are also displayed in **Table 1**. Although LS did not differ significantly between the two groups, TSI comparison yielded a large effect size (d=1.08), suggesting that the absence of statistical significance may reflect limited sample size rather than a true lack of difference. Liver stiffness values were similar between MASLD and CVID/AGA groups [7.4 (6.2–10.2) vs 7.1 (5.8–8.4)], whereas significant differences were observed in TAI [0.75(0.15) vs 0.61 (0.08)] and TSI [101.6 (7.67) vs 90.63 (7.89)] (**Table 2**).

**Table 2:** Comparison of patients with CVID and AGA and patients with MASLD.

	CVID+AGA	MASLD	*P-value*
Number of patients	31	42	
Age (median, IQR)	39 (30.5-56)	46.5 (40.25-57.75)	0.06
Male gender (%)	21 (68%)	29 (69%)	1.00
LS (median, IQR)	7.1 (5.8-8.4)	7.4 (6.2-10.2)	0.38
TAI (mean, SD)	0.61 (0.08)	0.75 (0.15)	**<0.001**
TSI (mean, SD)	90.63 (7.89)	101.6 (7.67)	**<0.001**

CVID-common variable immunodeficiency, AGA-agammaglobulinemia, MASLD- metabolic dysfunction-associated steatotic liver disease, SD-standard deviation, LS-liver stiffness measured by shear wave elastography, TAI-tissue attenuation imaging, TSI-tissue scatter imaging.

Statistically significant p-values and their corresponding correlation coefficients are highlighted in bold.

### 3.3 Biochemical profile and liver fibrosis-related biomarkers

Serum fibrosis markers were compared between the groups, as shown in **Table 1**. The ELF score ranged from 7.28 to 14.23, with a mean of 9.76 (± 1.36) in AGA and 9.23 (± 1.73) in CVID. According to ELF score categorization, 93.5% of patients were classified within fibrosis-associated categories, including 58% in the intermediate and 35.5% in categories associated with severe/advanced fibrosis. ELF score and analyzed biomarkers did not differ significantly between CVID and AGA patients (p=0.37).

After categorizing into four relevant groups (0=none/mild, 1=moderate fibrosis risk, 2=advanced fibrosis risk, and 3=cirrhosis risk), agreement between ELF and LS obtained by SWE was statistically significant (weighted Cohen’s kappa, κ=0.324; p=0.002).

Before adjusting for multiple comparisons, a statistically significant correlation was observed between LS assessed by SWE and the ELF score (r=0.5, p=0.01). Among analyzed individual fibrosis biomarkers, only HA showed positive correlation with SWE-derived LS (r=0.58, P = 0.003). ELF also showed statistically significant positive correlations with age (r=0.48, P = 0.006), AST (r=0.7, P< 0.001), ALT (r=0.42, P = 0.03), ALP (r=0.59, P = 0.003) and γGT levels (r=0.43, P = 0.04). HA levels were significantly correlated with age (r=0.56, P 0.001), ALT (r=0.49, P = 0.01), AST (r=0.75, P< 0.001), and γGT (r=0.57, P = 0.004).

Following adjustment for multiple comparisons, LS remained significantly associated with both ELF (P^adj^=0.05) and HA (P^adj^=0.03). ELF also retained significant association with age (P^adj^=0.04), AST (P^adj^<0.001) and ALP (P^adj^=0.03), whereas its correlations with ALT (P^adj^=0.13) and γGT (P^adj^=0.15) were lost. Similarly, HA remained significantly associated with age (P^adj^=0.02), ALT (P^adj^=0.05), AST (P^adj^<0.001) and γGT (P^adj^=0.04), but not with ALP (P^adj^=0.13).

On the other hand, TIMP-1 and PIIINP levels did not show any significant correlations with the other parameters investigated. No associations were observed between serum fibrosis markers and IgG, fibrinogen, or platelet count. These results, using adjusted P-values are summarized in **Table 3**.

**Table 3:** Correlation coefficients (s/p) between serum fibrosis markers, liver stiffness, and selected clinical and laboratory parameters in patients with CVID and AGA.

Parameter	ELF (s/p)	ELF *P/P^BH^*	TIMP-1 (s/p)	TIMP-1 *P/P^BH^*	PIIINP (s/p)	PIIINP *P/P^BH^*	HA (s/p)	HA *P/P^BH^*
LS	**0.50^s^**	**0.01/0.05^bh^**	0.02**^s^**	0.9/0.94^bh^	-0.18**^s^**	0.39/0.5^bh^	0.58^s^	**0.003/0.03^bh^**
TAI	0.45^p^	0.04/0.15^bh^	0.20**^s^**	0.36/0.48^bh^	-0.15**^s^**	0.52/0.62^bh^	0.21**^s^**	0.36/0.48^bh^
TSI	0.36^p^	0.11/0.27^bh^	-0.08**^s^**	0.72/0.8^bh^	0.25**^s^**	0.27/0.42^bh^	0.43^s^	0.05/0.16^bh^
Age	**0.48^s^**	**0.006/0.04^bh^**	0.00**^s^**	1/1^bh^	-0.02**^s^**	0.9/0.94^bh^	0.56^s^	**0.001/0.02^bh^**
ALT U/L	0.42^s^	0.03/0.13^bh^	0.10**^s^**	0.6/0.73^bh^	0.14**^s^**	0.49/0.6^bh^	0.49^s^	**0.01/0.05^bh^**
AST U/L	**0.70^s^**	**<0.001/<0.001^bh^**	0.23**^s^**	0.23/0.39^bh^	0.01**^s^**	0.93/0.95^bh^	0.75^s^	**<0.001/<0.001^bh^**
ALP U/L	**0.59^s^**	**0.003/0.03^bh^**	0.30^s^	0.15/0.31^bh^	0.32^s^	0.14/0.3^bh^	0.44^s^	0.03/0.132^bh^
γGT U/L	0.43^s^	0.04/0.15^bh^	0.07**^s^**	0.74/0.81^bh^	0.12**^s^**	0.59/0.69^bh^	0.57^s^	**0.004/0.04^bh^**
Fibrinogen g/L	-0.25^p^	0.3/0.44^bh^	0.02**^s^**	0.9/0.94^bh^	0.07**^s^**	0.75/0.81^bh^	-0.20**^s^**	0.38/0.49^bh^
IgG g/L	0.08**^s^**	0.65/0.77^bh^	-0.20**^s^**	0.3/0.44^bh^	0.07**^s^**	0.7/0.79^bh^	0.15**^s^**	0.44/0.56^bh^
Platelets x 10^9^/L	-0.03**^s^**	0.82/0.84^bh^	0.04**^s^**	0.81/0.83^bh^	/0.19**^s^**	0.32/0.45^bh^	-0.06**^s^**	0.73/0.8^bh^

Each parameter was independently correlated with ELF, TIMP-1, PIIINP, and HA; the corresponding P-values are shown for each correlation coefficient.

ELF-enhanced liver fibrosis score, TIMP-1-tissue inhibitor of matrix metalloproteinase, PIIINP-N-terminal pro-peptide of type III collagen, HA-hyaluronic acid, LS-liver stiffness measured by shear wave elastography, TAI-tissue attenuation imaging, TSI-tissue scatter imaging, ALT-alanine aminotransferase, AST-aspartate aminotransferase, ALP-alkaline phosphatase, γGT-gamma-glutamyl transferase, IgG-immunoglobulin G.

P^bh^ Benjamini–Hochberg adjusted P values for multiple comparisons,.

^p^Pearson’s correlation coefficient.

^s^Spearman’s correlation coefficient.

Statistically significant p-values and their corresponding correlation coefficients are highlighted in bold.

### 3.4 Subgroup analysis: CVID patients with and without enteropathy

Finally, comparison between CVID and CVID-E revealed significantly higher ALP values in the CVID-E group than in patients without enteropathy (102U/L in CVID-E vs 79U/L in CVID, P = 0.03). No significant differences were observed in serum fibrosis markers or LS measured by SWE, although ELF values tended to be higher in CVID-E (10.4 in CVID-E vs 9.39 in CVID, P = 0.09; d=0.73). In addition, CVID-E was associated with lower albumin (mean 41.1g/L vs 46.18g/L, P = 0.03; d=-1.09) and HDL levels (median 0.7mmol/L vs 1.09mmol/L, P = 0.02; r=-0.86). LDL levels were also lower in CVID-E (2.24mmol/L vs 3.31mmol/L), although with no significant differences (P = 0.2; r=-0.68). Finally, no difference in the incidence of splenomegaly was observed (37% vs 27%, P = 0.62, V = 0.11). **Table 4** summarizes the comparison between CVID and CVID-E patients.

**Table 4:** Comparison of CVID-E to CVID patients without enteropathy.

	Enteropathy (N = 8)	No enteropathy (N = 14)	*P-value*	Effect size (95% CI)
HA (median, IQR)	96.4 (45.4-139.3)	54.7 (28.7-77.2)	0.16	0.38 (-0.06-0.69)^r^
TIMP-1 (median, IQR)	116 (18.2-222)	119.6 (10.8-222.5)	0.87	-0.05 (-0.38-0.46)^r^
PIIINP (median, IQR)	27.3 (17-125.8)	17 (10.9-189.9)	0.62	0.14 (-0.30-0.53)^r^
ELF (mean, SD)	10.4 (1.8)	9.39 (0.89)	0.09	0.73 (-0.12-1.69)^d^
LS (median, IQR)	8.3 (5.9-13.35)	6.3 (5.7-7.85)	0.28	0.09 (-0.35-0.49)^r^
TAI (mean, SD)	0.64 (0.1)	0.6 (0.07)	0.40	0.45 (-0.39-1.37)^d^
TSI (mean, SD)	92.7 (6.2)	91.1 (9.24)	0.69	0.20 (-0.68-1.06)^d^
Age (median, IQR)	56 (44-58)	37 (30-60)	0.24	0.28 (-0.13-0.65)^r^
IgG g/L (median, IQR)	5.02 (3.62-9.2)	8.21 (5.93-9.56)	0.66	-0.13 (-0.52-0.31)^r^
AST U/L (median, IQR)	27 (26-35)	22 (17.7-29.25)	0.19	0.05 (-0.39-0.45)^r^
ALT U/L (median, IQR)	28 (24.5-29)	20 (14.75-25)	0.15	0.02 (-0.37-0.47)^r^
ALP U/L (median, IQR)	102 (94-127)	79 (70-103.25)	**0.03**	0.40 (-0.41-1.21)^r^
γGT U/L (median, IQR)	22 (18-25.5)	20 (16-34.5)	0.84	0.33 (-0.11-0.66)^r^
Platelets x 10^9^/L (median, IQR)	210 (169.75-270)	192 (147-316)	1.00	0.07 (-0.36-0.48)^r^
Fibrinogen g/L (mean, SD)	4.12 (0.87)	3.83(0.9)	0.56	0.33 (-0.55-1.20)^d^
Albumin g/L (mean, SD)	41.10 (5.81)	46.18 (3.1)	**0.03**	-1.09 (-2.14-(-0.25))^d^
HDL mmol/L (median, IQR)	0.7 (0.64-0.86)	1.09 (1.04-1.25)	**0.02**	-0.86 (-0.93-(-0.65))^r^
LDL mmol/L (median, IQR)	2.24 (1.44-2.95)	3.31 (2.37-3.47)	0.20	-0.68 (-0.85-(-0.33))^r^
Triglycerides mmol/L (median, IQR)	1.31 (1-1.63)	1.4 (1.16-1.6)	0.68	-0.40 (-0.70-(-0.02))^r^
INR (mean, SD)	1.16 (0.36)	1 (0.1)	0.25	0.60 (-0.19-1.60)^d^
Splenomegaly (%)	3 (37.5)	3 (27.2)	0.62	0.11 (-0.33-0.51)^v^

Effect sizes are presented as rank-biserial correlation (r) for Mann–Whitney U tests, Cohen’s d (d) for t-tests, and Cramér’s V (V) for categorical variables.

Abbreviations: 95% CI-95% confidence interval, HA-1 hyaluronic acid, IQR-interquartile range, TIMP-1-tissue inhibitor of matrix metalloproteinase-1, PIIINP-N-terminal pro-peptide of type III collagen, ELF-enhanced liver fibrosis score, SD-standard deviation, LS-liver stiffness measured by shear wave elastography, TAI-tissue attenuation imaging, TSI-tissue scatter imaging, IgG-immunoglobulin G, AST-aspartate aminotransferase, ALT-alanine aminotransferase, ALP-alkaline phosphatase, γGT-gamma-glutamyl transferase, HDL-high-density lipoprotein, LDL-low-density lipoprotein, INR-international normalized ratio.

Normal values: AST 0–37 U/L, ALT 0–41 U/L, ALP 40–120 U/L, gGT 0–49 U/L, albumin 34–55 g/L, fibrinogen 2.2-5.5 g/L, platelets 150-450x10^9^/L, IgG 5.5-16.3 g/L, HDL > 1 mmol/L, LDL 0-3.4 mmol/L, triglicerids 0-1.7 mmol/L.

Statistically significant p-values and their corresponding correlation coefficients are highlighted in bold.

## 4 Discussion

Liver disease in the context of CVID is frequently underrecognized, contributing to poor clinical outcomes and reduced survival (3–6, 13). The reported prevalence of hepatic involvement varies widely, largely depending on the definition of hepatic involvement and diagnostic approaches employed, with recent meta-analyses indicating a range between 9% and 79% (8). Although liver biopsy remains the gold standard for the diagnosis of NRH, granulomatous or autoimmune hepatitis, it carries procedural risks, and interobserver agreement is limited (10). Based on the available literature, LS assessments in patients with AGA have not been previously reported. These challenges underscore the critical need for reliable noninvasive biomarkers and diagnostic tools to improve the detection and monitoring of hepatic involvement in CVID and AGA. To our knowledge, this is the first study to evaluate the ECM biomarkers profiles and their relationship with SWE, integrating serum liver fibrosis-related biomarkers, the ELF score, LS, and additional tissue-related parameters, including TSI and TAI.

Recent literature supports the use of abdominal ultrasound as an initial imaging modality, which should be followed by TE of the liver (9, 29). Despite its growing use, no clear consensus exists regarding the optimal application of TE in CVID. In one study, LS was elevated (median 9.95 kPa) in patients with established CVID-L, and higher LS values were associated with the presence of portal hypertension (3). Conversely, another study reported no significant correlation between LS, hepatic venous pressure gradient, and histological fibrosis or elastosis scores (30). Nevertheless, a proposed LS cutoff of 11 kPa has been suggested as a threshold for considering liver biopsy (10). Taken together, these findings highlight both the potential utility and the current limitations of TE as a noninvasive tool for the assessment of hepatic involvement in CVID. It is important to note that previous studies have primarily focused on TE, a non-imaging technique, whereas our study employed SWE, which combines imaging with stiffness assessment and is considered more accurate than TE (31).

Although a considerable proportion of patients exhibited SWE values corresponding to fibrosis stages F1-F3 based on established cut-offs, these thresholds were originally derived from healthy individuals and cohorts with chronic hepatocellular liver disease (26, 32) and may not be directly applicable to CVID and AGA, in which hepatic involvement may reflect distinct underlying pathophysiological mechanisms. Interestingly, despite the absence of differences in LS between CVID/AGA patients and the MASLD comparator group, significant differences were observed in TSI and TAI parameters. These findings suggest that similar LS values may reflect distinct underlying pathophysiological processes, potentially driven by differences in ECM remodeling and immune-mediated tissue injury in primary antibody deficiencies compared with metabolic liver disease. In this context, SWE may be of particular relevance in CVID-L assessment, as it enables simultaneous evaluation of LS alongside additional tissue-related parameters such as TSI and TAI. This multiparametric approach, incorporating TSI and TAI, may capture microstructural and compositional tissue alterations not fully reflected by LS measurements alone, potentially indicating differences in parenchymal heterogeneity, ECM organization, vascular remodeling, and tissue attenuation characteristics. Collectively, these findings suggest that SWE may represent a valuable investigational approach for the assessment of hepatic involvement in the complex setting of CVID.

Both GLILD and liver disease are increasingly recognized as manifestations of immune dysregulation in CVID. Nearly half of patients with CVID-associated interstitial lung disease have been reported to exhibit concomitant liver disease (33). Furthermore, a recent study investigated the role of ECM remodeling markers, including MMP-7, MMP-9, and TIMP-1, in patients with GLILD in the context of CVID (20). To date, neither ELF nor its components (HA, TIMP-1, and PIIINP) have been evaluated for assessing hepatic involvement in CVID. Interestingly, the clinical applicability of the ELF score and its constituent biomarkers has been explored beyond liver pathology, including immune-mediated and autoimmune disorders, suggesting their potential relevance in diseases characterized by progressive chronic fibrosis (34–36).

In our study, LS measured by SWE correlated significantly with ELF score (P = 0.01; P^bh^=0.05) and HA (P = 0.003 P^bh^=0.03). Both ELF and HA were additionally correlated with age and liver enzymes (AST, ALP), indicating that these biomarkers may reflect cumulative hepatic injury. The observed correlation of HA with liver enzymes and age likely reflects its role as a marker of ECM remodeling in response to hepatocellular injury and chronic low-grade inflammation (37), both of which increase with advancing age and in chronic and immune-mediated disorders (38). Notably, TIMP-1 and PIIINP did not correlate with SWE or other biochemical parameters, highlighting that individual fibrosis markers may capture distinct aspects of ECM remodeling rather than a unified fibrotic process.

Agreement analysis using weighted Cohen’s kappa demonstrated concordance between ELF and SWE-based fibrosis staging, particularly within the intermediate fibrosis category. These findings suggest that the observed agreement is unlikely to be due to chance and indicate that ELF and SWE may reflect overlapping, yet non-identical, aspects of liver pathology.

However, these findings should be interpreted with caution, as both SWE-derived LS and ELF scores may be influenced by systemic immune activation and ECM turnover in CVID and AGA, thereby potentially overestimating the degree of true hepatic fibrosis. These methods should therefore be considered complementary rather than interchangeable.

Patients with CVID-E more frequently experience gastrointestinal (GI) infections and GI malignancies, while approximately 50% of these patients present with concomitant hepatic involvement (7, 24, 39). Both CVID-E and CVID-L share certain immunophenotypic and serological features, supporting the presence of common pathogenic mechanisms (40). Importantly, hepatopathy and severe enteropathy have been identified as major factors associated with premature mortality among patients with CVID complications of immune dysregulation (6). Considering these findings, we sought to investigate the extent of overlap between these two entities. Subgroup analysis within the CVID cohort did not demonstrate significant differences in fibrosis markers or LS between patients with and without enteropathy. Patients with CVID-E exhibited significantly lower albumin (P = 0.03) and higher ALP levels compared with non-enteropathic phenotype, suggesting subtle evidence of hepatic and biliary involvement, which may reflect chronic inflammatory burden, altered intestinal absorption, and early hepatobiliary dysfunction associated with enteropathy.

Notably, platelet counts remained within the normal range in our cohort. In the context of the known association between CVID-related liver disease, portal hypertension, hypersplenism, and thrombocytopenia, this observation supports the presence of predominantly early or subclinical hepatic involvement. Collectively, these observations suggest that subclinical hepatic involvement may be present in CVID, even in the absence of overt biochemical abnormalities or clinically diagnosed liver disease.

In our cohort, the ELF score ranged from 7.28 to 14.23. In comparison, the original ELF validation study reported ELF values ranging from 5.88 to 10.30 in 400 healthy individuals, with a reference interval of 6.72 (90% CI 6.58–6.84) to 9.79 (90% CI 9.45–10.01) (27). Overall, our CVID/AGA cohort showed a marked shift toward higher ELF values. Age has been previously identified as an important influencing factor for ELF values in healthy populations, with higher scores observed with increasing age, while modest intraday variability has also been reported (27). However, all samples in the present study were collected under standardized morning fasting conditions, thereby minimizing potential pre-analytical variability. Previous prospective studies have suggested that combining the ELF score with elastography-based techniques may improve the identification of patients with and without significant fibrosis and cirrhosis (41, 42). Furthermore, ELF appears to be more strongly influenced by inflammatory liver injury and less discriminative in low and moderate fibrosis stages (42). These observations warrant cautious interpretation of ELF values within intermediate fibrosis-associated categories, into which 58% of our patients were classified. Taken together, the ELF score may provide supportive evidence of hepatic involvement; however, its capacity to distinguish liver fibrosis from a broader pathogenic context of immune dysregulation in CVID, or from other underlying mechanisms in AGA, as well as its overall clinical applicability in these populations, requires further validation.

From a clinical perspective, these findings suggest that combining serum fibrosis-related biomarkers with SWE may help to identify CVID and AGA patients with potential hepatic involvement. However, this study is limited by its cross-sectional design and relatively small sample size, particularly in subgroup analyses. Although statistical significance was not reached for all comparisons, the magnitude of the observed effect sizes suggests that some between-group differences may still be clinically relevant and warrants further evaluation in larger cohorts. In addition, no internal healthy control group was included, although comparisons were based on validated biomarker reference ranges derived from large, published cohorts. Most importantly, the absence of histopathological confirmation represents a major limitation, as common patterns of CVID-related hepatic involvement, such as NRH and PSVD, may not be fully captured by fibrosis-oriented biomarkers and elastography thresholds derived from chronic hepatocellular liver diseases. Furthermore, ELF and SWE have not been formally validated in patients with CVID or AGA, warranting cautious interpretation of fibrosis staging based on these modalities. Therefore, the findings should be considered exploratory and hypothesis-generating, requiring validation in larger prospective studies.

Despite these limitations, our findings provide supportive evidence of subclinical hepatic involvement in CVID and AGA and emphasize the need for longitudinal studies to determine the prognostic values of these biomarkers, and to define disease-specific thresholds.

## 5 Conclusion

In conclusion, our exploratory findings suggest subclinical hepatic involvement in CVID and AGA. The combination of ELF score and SWE may represent a promising non-invasive approach to liver assessment, capturing distinct but complementary aspects of hepatic involvement. To our knowledge, this is the first study to integrate SWE with serum liver fibrosis-related biomarkers and to examine their relationship in this patient population. The associations observed between ELF, particularly its hyaluronic acid component, and SWE-derived LS suggest a possible link between extracellular matrix remodeling and hepatic involvement in primary antibody deficiencies. These findings should, however, be interpreted with caution, as both ELF and SWE-derived LS may be affected by systemic immune activation and disease-related alterations, potentially limiting their specificity for true hepatic fibrosis. Accordingly, larger, prospective, multicenter studies with histopathological correlation are needed to validate their clinical utility and define disease-specific thresholds.

## Data Availability

The original contributions presented in the study are included in the article/**Supplementary Material**. Further inquiries can be directed to the corresponding author.

## Supplementary material

Supplementary Figure 1 Scatter plots representing correlation analysis between elastography parameters and ELF score. Picture **(A)**-Correlation between ELF score (x-axis) and liver stiffness (kPa) measured by shear wave elastography (y-axis). Picture **(B)**-Correlation between TAI (x-axis) and ELF score (y-axis). Picture **(C)**-Correlation between TSI (x-axis) and ELFscore (y-axis). Correlation coefficients were calculated using Spearman’s rank test for picture **(A)** and Pearson’s test for pictures **(B, C)**. Correlation coefficients and corresponding p-values are reported in the Results section and **Table 3**. ELF-enhanced liver fibrosis score, TAI-tissue attenuation imaging, TSI-tissue scatter imaging.
