# Supplementary figures and images for: Non-invasive assessment of hepatic involvement in common variable immunodeficiency and agammaglobulinemia using enhanced liver fibrosis score and shearwave elastography
Source: Front Immunol. 2026 Jun 24;17:1857089. doi: 10.3389/fimmu.2026.1857089 (PMC13341503; doi:10.3389/fimmu.2026.1857089)

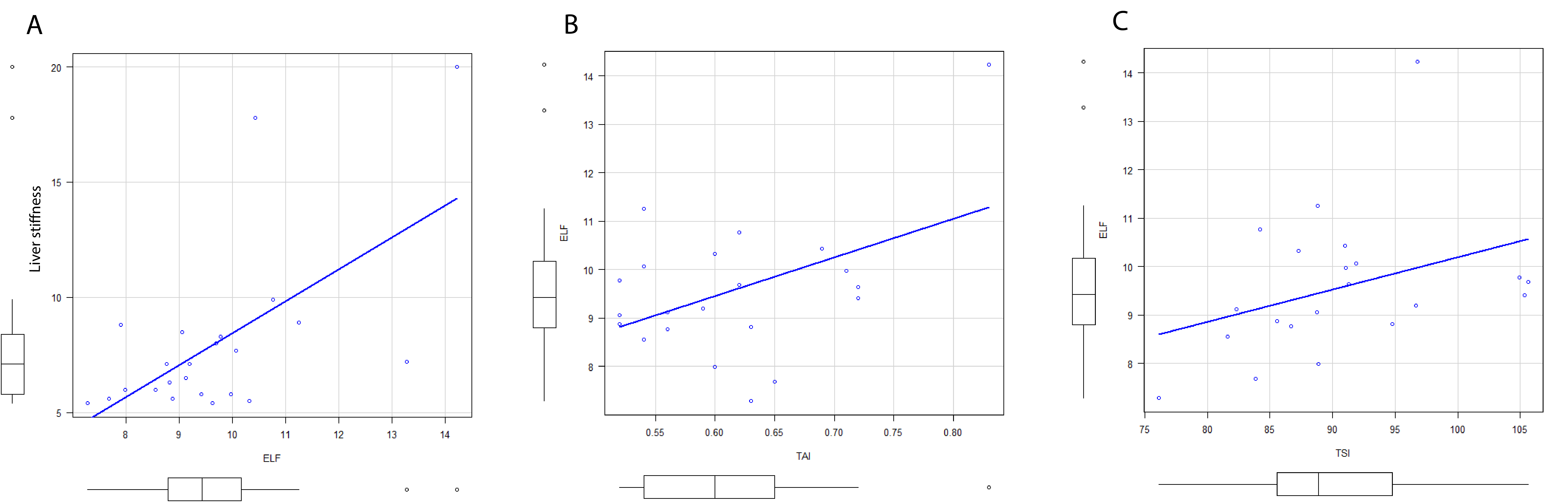

Supplement: Supplementary Figure 1 — Scatter plots representing correlation analysis between elastography parameters and ELF score. Picture (A)-Correlation between ELF score (x-axis) and liver stiffness (kPa) measured by shear wave elastography (y-axis). Picture (B)-Correlation between TAI (x-axis) and ELF score (y-axis). Picture (C)-Correlation between TSI (x-axis) and ELFscore (y-axis). Correlation coefficients were calculated using Spearman’s rank test for picture (A) and Pearson’s test for pictures (B, C). Correlation coefficients and corresponding p-values are reported in the Results section and Table 3. ELF-enhanced liver fibrosis score, TAI-tissue attenuation imaging, TSI-tissue scatter imaging. [file Image1.png]
